# Spatial working memory is critical for gesture processing: Evidence from gestures with varying semantic links to speech

**DOI:** 10.3758/s13423-025-02642-4

**Published:** 2025-02-10

**Authors:** Demet Özer, Aslı Özyürek, Tilbe Göksun

**Affiliations:** 1 Department of Psychology, Bilkent University, Ankara, Türkiye; 2 Department of Psychology, Koç University, Istanbul, Türkiye; 3 Max Planck Institute for Psycholinguistics, Nijmegen, Netherlands

**Keywords:** Spatial skills, Verbal skills, Gesture processing, Semantic relation of gesture

## Abstract

Gestures express redundant or complementary information to speech they accompany by depicting visual and spatial features of referents. In doing so, they recruit both spatial and verbal cognitive resources that underpin the processing of visual semantic information and its integration with speech. The relation between spatial and verbal skills and gesture comprehension, where gestures may serve different roles in relation to speech is yet to be explored. This study examined the role of spatial and verbal skills in processing gestures that expressed redundant or complementary information to speech during the comprehension of spatial relations between objects. Turkish-speaking adults (*N*=74) watched videos describing the spatial location of objects that involved perspective-taking (left-right) or not (on-under) with speech and gesture. Gestures either conveyed redundant information to speech (e.g., saying and gesturing *“left”*) or complemented the accompanying demonstrative in speech (e.g., saying “*here,*” gesturing *“left”*). We also measured participants’ spatial (the Corsi block span and the mental rotation tasks) and verbal skills (the digit span task). Our results revealed nuanced interactions between these skills and spatial language comprehension, depending on the modality in which the information was expressed. One insight emerged prominently. Spatial skills, particularly spatial working memory capacity, were related to enhanced comprehension of visual semantic information conveyed through gestures especially when this information was not present in the accompanying speech. This study highlights the critical role of spatial working memory in gesture processing and underscores the importance of examining the interplay among cognitive and contextual factors to understand the complex dynamics of multimodal language.

**Supplementary information:**

The online version contains supplementary material available at 10.3758/s13423-025-02642-4.

Human communication is a joint coordinated activity whereby interlocutors exchange several multimodal signals such as speech and *co-speech representational hand gestures* (henceforth, *gestures*^1^). These gestures are spontaneous hand movements that co-occur with relevant segments of the accompanying speech and represent events, object attributes, or spatial locations (McNeill, 1992). Gestures can convey redundant information to accompanying speech or additional information that complements the co-occurring speech (Krauss et al., 1996). Listeners integrate the information they see in such gestures together with speech (Kelly et al., 2010). Gestures facilitate language comprehension and learning, particularly for visual-spatial information (e.g., Beattie & Shovelton, 1999; Dargue & Sweller, 2020). Yet recent research also suggests that gestures do not lead to improved comprehension in all instances and for all individuals (Dargue et al., 2019; Özer & Göksun, 2020a). The effects of gestures on language comprehension might show variations dependent on contextual factors associated with the properties of gestures, such as the semantic relation of gestures to the accompanying speech (e.g., Dargue et al., 2021) or on cognitive factors, such as individuals’ working memory capacities (e.g., Özer & Göksun, 2020b).

Extending previous research, the current study examines how these two factors (i.e., semantic relation and cognitive skills) interact together to yield different outcomes for gesture processing. Specifically, we investigate whether and how listeners’ spatial and verbal skills relate to their ability to comprehend spatial information across different language contexts, including unimodal utterances, where either speech or gesture serves as the primary source of information, and bimodal utterances, where speech and gesture are used together with gestures typically expressing redundant information to speech. In unimodal utterances with gesture-only information, gestures typically express critical spatial information that complements the accompanying speech. Our objective is to provide a more nuanced understanding of multimodal communication by unveiling the interplay of the contextual and cognitive factors contributing to the effects of gestures on comprehension.

## Spatial and verbal skills for the role of gestures in language comprehension

*Visual-spatial skills* are critical for semantic uptake and processing of gestures (Kelly & Goldsmith, 2004; Wu & Coulson, 2014). Gestures are visual articulators, conveying analog visual information through continuous dynamic events in the space. Given that gestures represent meaning in the visual-spatial medium, visual-spatial cognitive resources are required to interpret and maintain visual semantic information conveyed through gestures for the subsequent integration with the accompanying speech (Arslan et al., 2024; Momsen et al., 2021; Özer & Göksun, 2020a; Wu et al., 2022). However, *verbal skills* might also play a role in gesture processing (Momsen et al., 2020; Wagner et al., 2004) as speech and gesture are closely linked and experienced mostly together. Gestures are part of the language system and used in coordination with the speech. Indeed, the neural instantiation of processing gestures parallels the processing of verbal information (Özyürek, 2014; Straube et al., 2012; Xu et al., 2009). Developmental work also suggests that children benefit more from gestures as their verbal skills mature by age (Kartalkanat & Göksun, 2020) and verbal skills (i.e., receptive vocabulary) predict children’s gesture comprehension (Doğan et al., 2024), indicating the important role of verbal abilities in gesture processing.

If visual-spatial and verbal skills are linked with processing gestures, individual differences in these skills might lead to variation in how and to what extent listeners process and benefit from gestures (Özer & Göksun, 2020a). Özer and Göksun (2020b) examined how spatial and verbal working memory (WM) capacities related to processing gesture-speech utterances in a mismatch paradigm in which either gesture or speech conveyed a mismatching^2^ information to a preceding action prime video (Kelly et al., 2010). Participants processed mismatching information in relation to a preceding action prime (e.g., *cutting* paper), either in the visual modality (*gesture-mismatch*, e.g., saying “*cut*” and gesturing “*turn*”) or in the auditory-verbal modality (*speech-mismatch*, e.g., saying “*turn*” and gesturing “*cut*”). They found a modality-specific link between spatial versus verbal WM capacities and processing information from the corresponding channel during multimodal language comprehension. Listeners’ spatial WM capacities were associated with increased performance only for the gesture-mismatch condition, whereas their verbal WM capacities were associated with increased performance only for the speech-mismatch condition. In a similar vein, spatial WM has been shown to be associated with better gesture processing (Momsen et al., 2021; Wu & Coulson, 2014) and greater benefits from observing gestures during comprehension and learning (Aldugom et al., 2020; Brucker et al., 2022). This evidence suggests that spatial WM plays a prominent role in the interpretation and maintenance of visual information conveyed through gestures. However, van Wermeskerken et al. (2016) reported no relation between spatial WM capacity and the enhancing effects of observing gestures on learning. On the other hand, evidence so far showed either no effect of verbal WM capacity on processing co-speech gestures (Aldugom et al., 2020; Özer & Göksun, 2020b; Wu & Coulson, 2014) or an effect of verbal WM to interpret gestures whose referents were ambiguous (Momsen et al., 2020).

Gestures exhibit varying semantic relationships with speech: *redundant gestures* reiterate information already expressed in the accompanying speech, while *complementary gestures* provide additional information beyond that conveyed by speech. It is unknown whether and how visual and verbal skills affect processing of co-speech gestures when the gesture’s semantic content differs from that of the co-speech.

### Semantic relations between gesture and speech

Speakers commonly employ complementary gestures, particularly when conveying visual-spatial information (e.g., describing the relative positions of objects), as gestures excel at expressing such information due to their representational capabilities (Beattie & Shovelton, 2006; Goldin-Meadow, 2003). For instance, a speaker might use a pointing or iconic gesture to illustrate an object’s location, along with a demonstrative in their speech, such as “here” (Cooperrider, 2016, 2017; Emmorey & Casey, 2001; Peeters & Özyürek, 2016; Slonimska et al., 2015). Earlier work suggested that gestures that convey additional complementary information crucial for successful comprehension are more beneficial compared with gestures that express redundant information that is already expressed in speech (Dargue et al., 2021; Hostetter, 2011; Yeo et al., 2017). However, it is important to note that “complementary gestures” in earlier studies provided additional information in narrative contexts that specifies and/or enrich details that are not directly articulated in speech (e.g., raising one finger while saying, “he had won a prize,” rather than explicitly stating that he had won the first prize; Dargue et al., 2021). Gestures that complement the spoken deictic terms in speech (e.g., demonstratives such as “here”) during descriptions of spatial relations, on the other hand, convey the spatial information exclusively through the visual modality. These differences in the conceptualization of complementary gestures across earlier work and the current study may lead to differential patterns of language comprehension outcomes and underscore the need to further examine the role of gestures across varying contexts.

Listeners tend to direct more visual attention to gestures that complement demonstratives in speech compared with those that merely duplicate information, indicating that complementary gestures are subject to increased visual processing driven by the speech context (Özer et al., 2023). Additionally, gestures that help to disambiguate degraded speech get more direct visual attention, particularly for nonnative listeners with lower verbal proficiencies (Drijvers et al., 2019). Furthermore, brain regions responsible for processing combinations of speech and gesture, such as the left inferior frontal gyrus and middle temporal regions, show increased activity when processing complementary gestures as opposed to redundant ones (Demir-Lira et al., 2018; Dick et al., 2014). These findings suggest that behavioral and neural processing of gestures that express redundant versus complementary semantic information show differentiation.

Spatial and verbal skills can distinctly affect the processing of redundant versus complementary gestures. Spatial skills might be more important for comprehending complementary gestures, as the semantic information required for the successful comprehension of the message can only be discerned through gestures in those instances. Since complementary gestures often convey visual-spatial information that augments the accompanying speech, individuals with higher spatial skills might excel in processing complementary visual information, enhancing their overall language comprehension. Redundant gestures, on the other hand, might rely less on spatial and more on verbal skills as they reiterate the information already conveyed in speech.

### The present study

In this study, we asked whether and how listeners’ *spatial* and *verbal skills* are related to observing gestures expressing *redundant versus complementary* information to speech during the comprehension of relative spatial locations of objects. We examined two types of spatial relations: *viewpoint-dependent (left-right)* spatial relations that require viewpoint alignment between interlocutors and *viewpoint-independent (on-under)* spatial relations that are void of such alignment. Earlier work mostly focused on observing gestures in comprehending viewpoint-independent spatial relations (Beattie & Shovelton, 2006; Holler et al., 2009). Yet observing left-right gestures from the speakers’ egocentric perspective creates more processing load than for viewpoint-independent relations as listeners need to switch spatial perspectives (Hostetter et al., 2018). Although spatial skills remain important for processing of both viewpoint-dependent and viewpoint-independent spatial relations in speech and gestures, they might be particularly essential in the context of left-right relations, which require spatial perspective-taking with heightened cognitive demands.

**Fig. 1:**
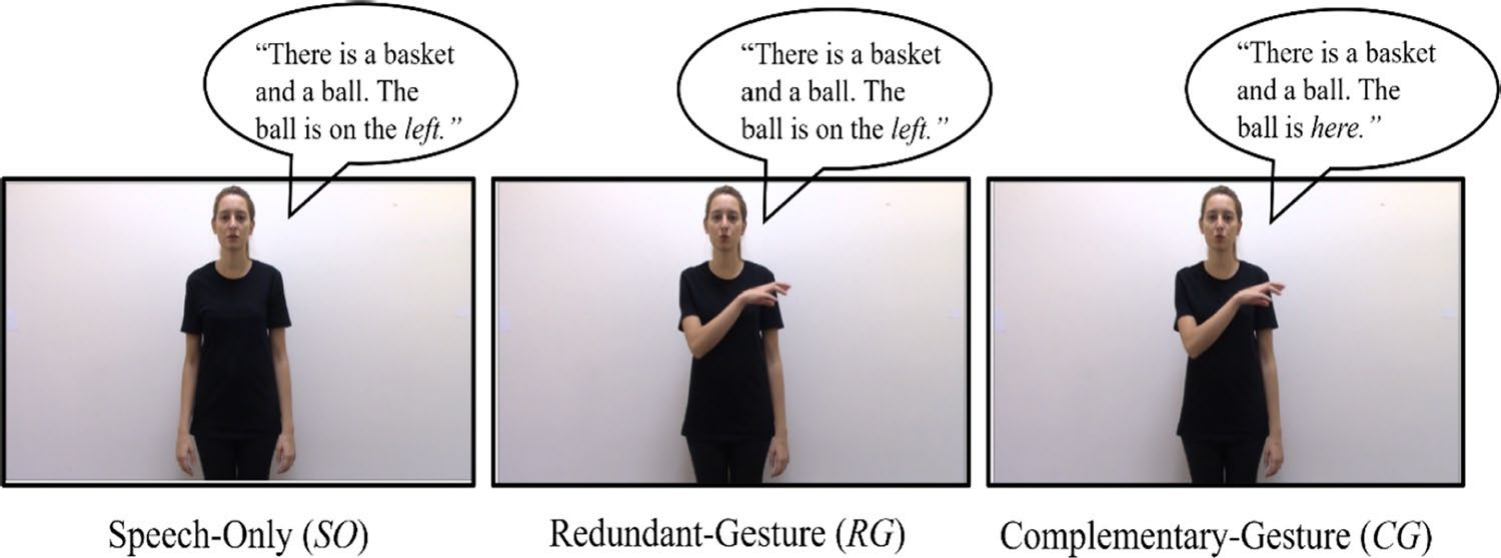
Three conditions in the experimental task. Although written here in English, the original stimuli were in Turkish. The underlined word denotes the speech that the gesture temporally overlaps with

We asked native Turkish-speaking adults to watch videos of an actress who described *left-right* versus *on-under* relations between two objects in three conditions (see Fig. 1). The relative location of the objects was provided (1) only in speech (speech-only [SO]), (2) both in speech and gesture (redundant-gesture [RG]), or (3) only in gesture coupled with a demonstrative (“here”; complementary-gesture [CG]). We measured accuracy and reaction times (RT) as participants chose the picture depicting the described spatial relation among four alternatives. For spatial skills, we used the Corsi block span task (visual-spatial WM capacity) and the mental rotation task (overall spatial ability). For verbal skills, we used the digit span task that measures verbal WM capacity.

We employed two spatial tasks that assessed distinct aspects of spatial skills. The Corsi block span task measures visual-spatial working memory (WM) capacity, which is generally associated with the maintenance of visual-spatial information. In contrast, the mental rotation task measures overall spatial ability, primarily tapping into intrinsic (i.e., within-object) dynamic abilities which feature the representation and manipulation of transformation and movement (Hodgkiss et al., 2018; Kozhevnikov & Hegarty, 2001; Newcombe & Shipley, 2015). Although both tasks serve as measures of spatial skills and are often correlated, they capture different underlying spatial mechanisms and abilities. Previous research has demonstrated the influence of visual-spatial WM capacity (e.g., Corsi block span task in Özer & Göksun, 2020b; Wu & Coulson, 2014; Wu et al., 2022; and visual patterns task in Aldugom et al., 2020) on gesture comprehension. Building on this evidence, we employed the Corsi block span task in the present study. However, as the experimental task in our study involves processing relative spatial relations between objects, which is an ability that relies on dynamic object-related spatial skills, we also included the mental rotation task to explore whether and how general spatial skills pertaining to intrinsic object-related spatial skills influence gesture processing, particularly in a context that require such skills. The digit span task was selected as a measure of verbal working memory to ensure consistency in terms of task structure with the Corsi block span task; both are simple working memory tasks designed to measure the maintenance of information across spatial versus verbal domains. This parallel structure allowed us to examine the role of spatial and verbal skills across comparable cognitive loads, facilitating a clearer interpretation of their roles in gesture processing.

Our predictions were as follows.

(i) *Task performance:* Listeners’ comprehension gets facilitated for bimodal utterances (gesture + speech) compared with unimodal ones (speech-only or gesture-only, e.g., Beattie & Shovelton, 1999; Dargue & Sweller, 2020). If observing gestures that are produced along with redundant speech improves comprehension compared with unimodal utterances that only include speech, participants would be more accurate and faster in the RG than in the SO condition. For the comparison between two unimodal conditions (i.e., when the critical spatial information was expressed solely either in speech or in gesture), we expected the performance in the SO to be higher compared with the CG, as it would be easier to discern the categorical spatial relation information from the speech compared with the gesture. This prediction was also based on the evidence indicating “verbal bias”—speech tends to be a more prominent channel on which listeners rely, even in the face of contradicting information (Arslan et al., 2024). Participants would also be more accurate and faster in all conditions for on-under than for left-right trials that require spatial perspective-taking.
(ii) *Spatial skills and task performance:* Both the Corsi block span task and the mental rotation task would relate to higher accuracies and shorter RTs when there was a gesture (RG and CG). However, this association would be more pronounced for the CG than for the RG, as the location information was given only in the visual-spatial modality in the CG. Performance in both tasks would also be particularly related to better performance for left-right relations that required spatial perspective-taking compared with on-under trials.
(iii) *Verbal skills and task performance:* The digit span task would be related to higher accuracies and shorter RTs only when the location information was provided through speech with or without redundant gesture (SO and the RG).

## Method

### Participants

We recruited 74 native Turkish-speaking participants (49 women, *M*_*age*_ = 21 years) from Koç University, Istanbul, in return for either course credit or monetary compensation. Four participants were discarded: three participants due to experimenter error, and one participant reported misunderstanding the instructions for the experimental task. The final sample consisted of 70 participants (48 women, *M*_*age*_ = 21.1 years, *SD*_*age*_ = 3.1, age range: 18–35; *M*_*education*_ = 15.3 years, education range: 12–25). All participants were right-handed, had normal or corrected-to-normal vision, and had no hearing impairments. All participants gave informed consent before the testing, which was approved by the Ethics Committee on Human Research of Koç University (Protocol Number: 2019.172.IRB3.102).

### Stimuli and materials

#### The experimental task

The experimental task consisted of short video clips of an actress who described two types of spatial relations between a figure and a ground object in different displays: viewpoint-dependent spatial relations (*left-right*) and viewpoint-independent spatial relations (*on-under*). The actress described the spatial location of the figure object in relation to the ground object in three conditions (see Fig. 1). These conditions were (1) speech-only (SO): The actress conveyed the relative spatial position of the figure object only in speech by using the appropriate preposition without making any gesture (e.g., the actress said, “There is a basket and a ball. The ball is on the *left*” while standing still); (2) redundant-gesture (RG): The actress expressed the spatial location in both speech and gesture by using the appropriate preposition in her speech and showing the location of the figure object with her gesture (e.g., the actress said “There is a basket and a ball. The ball is on the *left*” while gesturing to her left-hand side); and (3) complementary-gesture (CG): The actress conveyed the spatial location of the figure object with her gesture that complements the accompanying demonstrative in speech (“here”; e.g., the actress said “There is a basket and a ball. The ball is *here*” while gesturing to her left-hand side).

**Fig. 2:**
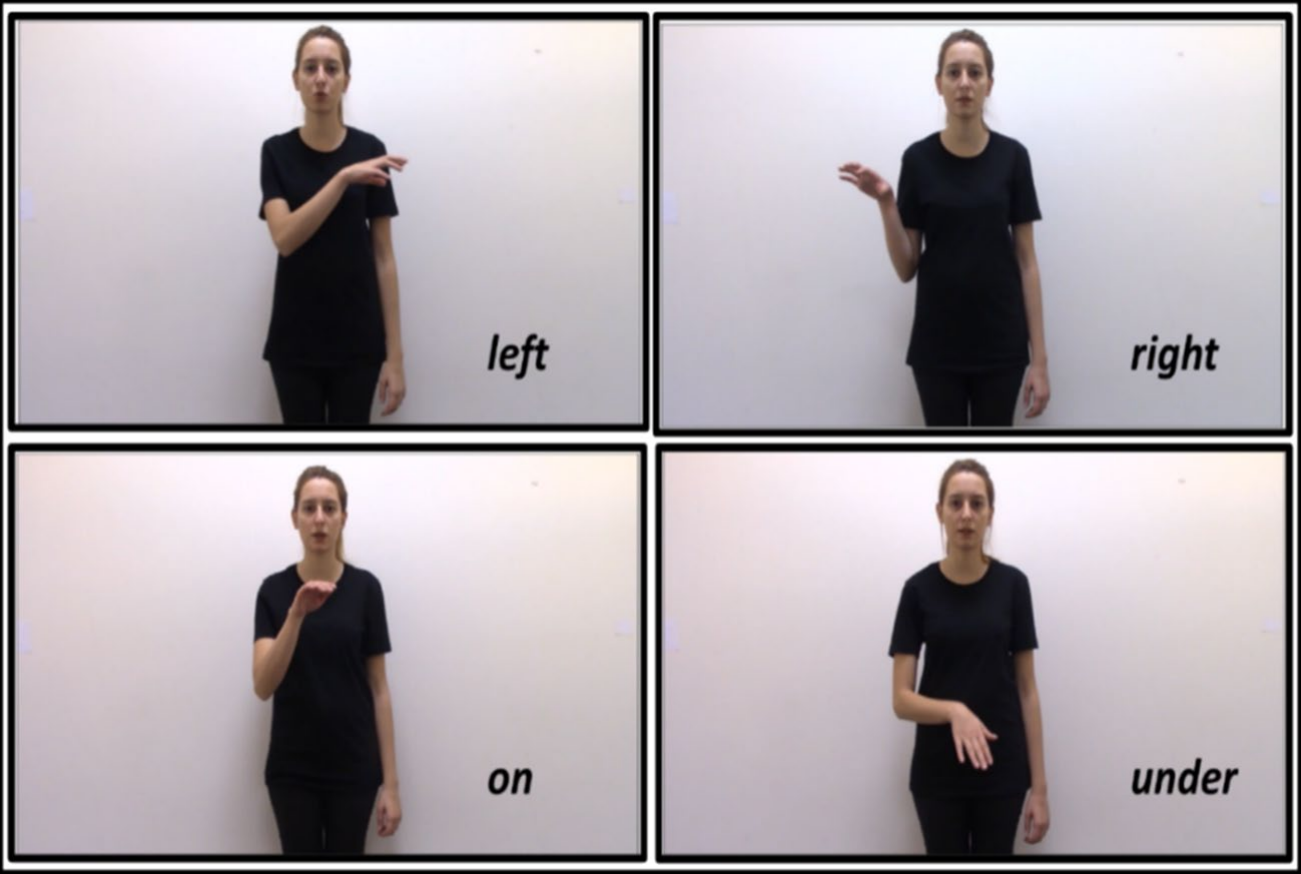
Left-right and on-under (Spatial prepositions of “on” and “above” in English are referred by the same word of “üst” in Turkish.) gestures that were used in the experimental task

In each clip, the actress produced two sentences. The first sentence introduced the ground object and the figure object in order (e.g., “there is a basket [GR] and a ball [FIG]”), and the second sentence presented the relative spatial location of the figure in relation to the ground object without naming the ground (e.g., “the ball [FIG] is on the *left (sol) / right (sağ) / on (üst) / under (alt)/ here (burda)*”). The way we developed the speech stimuli was based on an earlier study, where Turkish-speaking adults spontaneously described left-right and on-under relations between a central ground object and a figure object, similar to the displays used in the present study (Karadöller, 2022). Turkish speakers typically introduced the ground object first (e.g., “There is a basket”) followed by the figure object (e.g., “and a ball”). Then, they tend to describe the relative spatial relation of the figure relative to the ground, often omitting the explicit mention of the ground in the latter part of their description (e.g., “The ball is on the left”; Karadöller, 2022). We designed the speech in the current study in accordance with this observed pattern seen in spontaneous descriptions of native Turkish-speakers to ensure that our stimuli are as naturalistic and ecologically valid as possible (see Özer et al., 2023, for further details). During the second sentence, the actress made a gesture with her right hand (palm-down) as she uttered the spatial relation term. For left-right gestures, the actress extended her right arm to the left and right sides of her torso, respectively. For on-under gestures, the actress moved her right hand slightly towards the face and slightly towards the feet, respectively. Figure 2 presents the left-right and the on-under gestures used in the experimental paradigm.

All videos displayed the actress from the head to the knees, appearing in the same starting position (i.e., in the middle of the screen) with hands casually hanging on each side of the body. After making a gesture, the actress retracted her hand to its initial position. The actress wore black clothes, and the background was white. For the videos in which the actress made a gesture (i.e., the RG and CG), we inserted the audio files of their SO counterparts to control for the possible confounding effects of prosodic prominence that might be affected by gesturing (Krahmer & Swerts, 2007). The videos were 5-s long (see Özer et al., 2023, for a similar procedure). Examples of video stimuli are available in the Open Science Framework repository (view-only link here: https://osf.io/d6xzw/?view_only=be16d258186b4b4aa1296df30b866926). Please contact the corresponding author for the entire set of stimuli.

#### Individual differences measures

##### Corsi block span task

We used the computerized version of the forward Corsi block tapping task (Kessels et al., 2000) to measure spatial working memory capacity, which was originally developed by Corsi (1972). In this task, participants were presented with an asymmetric array of nine blue squares on the screen. In each trial, some of the squares flashed in sequence. After the flashings ended, the participants were instructed to click each square in the same order that the flashes occurred. Sequences started from three and proceeded to nine, with two chances at each sequence length. Participants advanced to the next level by entering the flashes in one sequence correctly. We measured the block span, which is the length of the last correctly recalled sequence.

##### Mental rotation task

We used the computerized version of the mental rotation task (Ganis & Kievit, 2015) to assess visual-spatial skills (adapted from Shepard & Metzler, 1971). In this task, participants were presented with two 3-dimensional cube objects side-by-side on the screen. The cube objects were rotated by 0, 50, 100, or 150° on each trial. Participants were instructed to mentally rotate the objects and decide whether they were the same or the mirror images of each other (i.e., different). We measured the percentage of correct responses across all trials.

##### Digit span task

We used the computerized version of the auditory forward digit span task (Woods et al., 2011, originally adapted from Wechsler, 1939) to measure verbal working memory capacity. In this task, participants listened to a sequence of digits, spoken one at a time, at the rate of one digit per 2 s. At the end of the sequence, participants were instructed to select the digits in the order they listened to them among digits displayed in a circular array on the computer screen. The task started with three sequences of digits and proceeded to 14, moving up a level after two consecutive correctly recalled sequences. We measured the digit span, which is the maximum number of digits recalled correctly, before making two consecutive errors.

### Procedure

**Fig. 3:**
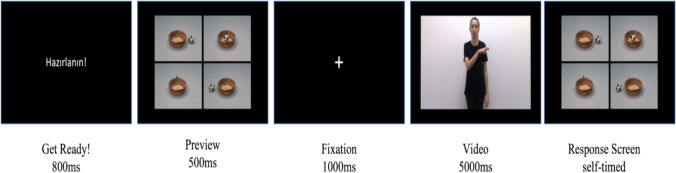
A single trial in the experimental task

Participants were tested in a dimly lit soundproof room on a 17-in. Acer laptop. After they gave informed consent and filled out the demographics form, they completed the experimental task. In this task, they watched videos of an actress describing different spatial relations between two objects. After watching the video, they were presented with the response screen, on which they selected the picture that best depicted the spatial relation described in the preceding video among four alternatives. In the response screen, there were four pictures depicting different spatial relations between the same figure and the ground objects (see “response screen” in Fig. 3). In each picture, the ground object was always at the center, and the relative position of the figure object in relation to the ground object changed. They were instructed to select pictures by clicking them with the mouse as fast and accurately as possible.

A single trial proceeded as follows: a “Get Ready!” screen for 800 ms, a preview of the response screen for 500 ms, a fixation cross for 1,000 ms, the video for 5,000 ms, followed by a response screen, which lasted until participants gave a response (see Fig. 3). There were 10 trials for each combination of the condition and the spatial relation, making 150 trials in total: 10 trials × 3 conditions (SO, RG, CG) × 5 spatial relations (left, right, on, under, in). We used trials with the spatial relation of “*in*” as fillers to introduce variability to the spatial relations observed in the experimental task. By doing so, we aimed to minimize potential learning and strategy development that could arise from the repeated exposure of left-right and on-under trials. Therefore, out of 150 trials, 120 were experimental trials, and 30 were filler trials. All trials were presented in random order on E-Prime 3.0.

Participants went through familiarization and practice trials before they completed the experimental task. First, they saw seven pictures, each of which depicted a different spatial relation between two objects and a written label for the spatial relation (i.e., left, right, on, under, front, behind, in). Although front-behind trials were not used in the task, we included them in this phase to make participants familiar with the general notion of spatial relations. Second, the experimenter demonstrated how to complete the task with five sample videos from the RG condition (one for each spatial relation used in the task: left-right, on-under, and in). Last, they completed 12 practice trials (four from each condition) and were given feedback by the experimenter if they gave incorrect responses. After the training, the experimenter reminded participants to respond as fast and accurately as possible. Participants completed the rest of the task individually. The experimental task lasted around 30 min.

After the experimental task, participants completed individual differences tasks in the fixed order: Corsi block tapping task, auditory digit span task, and mental rotation task. For the Corsi block tapping task and the digit span task, the experimenter explained how to complete the tasks without any demonstration and training. For the mental rotation task, participants completed 15 practice trials with written feedback (correct-incorrect). There were 96 trials in total: 12 different cube objects × 4 rotation angles × 2 sameness category. Participants got no feedback during the experimental trials. All individual differences tasks were implemented on Inquisit 5 and lasted around 15 min. After the completion of the individual differences task, participants were debriefed and thanked for their participation. The entire procedure lasted around 45 min.

### Analyses

There were 8,400 observations in total (120 experimental trials per participant × 70 participants). For RTs, we discarded trials that were two standard deviations above or below the mean (*N* = 310 trials) and incorrect responses (*N* = 531 trials), leaving 7,559 trials in total for RT analyses. Next, we discarded individual differences scores that were three standard deviations above or below the sample mean. There were no outliers for the Corsi block tapping and the digit span tasks. We discarded the score of one participant (12% accuracy across the entire task and the acceptable range was 0.79 ± 0.29) from further analyses that incorporated the term of mental rotation scores. The final number of observations for each analysis is as follows. For accuracy-related analyses: 8,400 observations for Corsi block span and digit span scores and 8,280 observations for mental rotation scores. For RT-related analyses: 7,559 observations for Corsi block span and digit span scores and 7,445 for mental rotation scores.

We used linear mixed-effects regression models in four different sets of models. Each model was run separately for accuracy (binary) and RT (continuous) as outcome variables. We used the logistic version of the model for the accuracy. In Set 1, we asked whether and how accuracy (Model 1a) and RT (Model 1b) changed across conditions and spatial relation (SR) types. The fixed effects included condition, SR type, and two-way interaction between the two. In Set 2 to Set 4, we asked whether and how Corsi spans (Models 2a and 2b), mental rotation scores (Models 3a and 3b), and digit spans (Models 4a and 4b) were associated with accuracy and RT across conditions and SR types. For each model, the fixed effects included the corresponding individual differences measure, condition, SR type, and all two- and three-way interactions among them. In all models, the random effects included the random subject and item intercepts.

We performed all analyses with the *lme4* package (Bates et al., 2015) on RStudio (RStudio Team, 2020). In generalized (i.e., logistic) models, we used the “bobyqa” optimizer, which maximized the number of iterations performed in a model to alleviate possible convergence problems (Powell, 2009). All continuous predictor variables were scaled and centered on the mean (*M* = 0, *SD* = 1). There were significant positive bivariate correlations among all continuous predictors (see Supplementary Materials), yet multicollinearity did not pose any problem for our analyses as predictor variables were used in separate models. For the models with RT as the continuous outcome variable, we used the log transformation of RT scores to avoid problems associated with skewed data. We set the contrast coding of all categorical variables to sum-to-zero, which means that the intercept corresponded to the grand mean and each contrast encoded the deviation from the intercept for a given factor. We used the *car* package (Fox & Weisberg, 2019) to obtain Type III Wald Chi-square test results, which showed whether the inclusion of each term in a model (i.e., explanatory variables) significantly improved the model. We used the *interactions* package (Long, 2019) to investigate interactions. We reported Bonferroni-adjusted pairwise comparisons within and across the categorical variables using the *emmeans* function, and simple slope estimates for interactions with continuous predictors using the *sim_slopes* function. We used the *ggplot2* (Wickham, 2016) and *jtools* (Long, 2020) packages for data visualization. The R code and datasets are available in the Open Science Framework repository (view-only link here: https://osf.io/d6xzw/?view_only=be16d258186b4b4aa1296df30b866926).

## Results

We report only significant effects and interactions here. Descriptive statistics for the measures of individual differences and the full set of results for mixed models can be found in the Supplementary Materials.

### Set 1. Task performance across conditions and spatial relation (SR) types

#### Accuracy (Model 1a, Fig. 4)

There was a main effect of the condition, ${\chi }^{2}$(2) = 82.87, *p* < .001. Participants were less accurate in the CG compared with the RG (*β* = 1.35, *SE* = 0.18, *z* = 7.49, *p* < .001) and to the SO (*β* = 1.42, *SE* = 0.18, *z* = 7.77, *p* < .001). There was no difference between the SO and the RG (*p* > .05). The effect of the spatial relation (SR) type on accuracy was qualified by an interaction with the condition, ${\chi }^{2}$(2) = 6.43, *p* = .04. Participants were less accurate for on-under trials compared with left-right trials only in the CG (*β* = 0.59, *SE* = 0.23, *z* = 2.55, *p* = .01). There was no difference between left-right and on-under trials in the SO and the RG (*p *values > .05)*.*

**Fig. 4:**
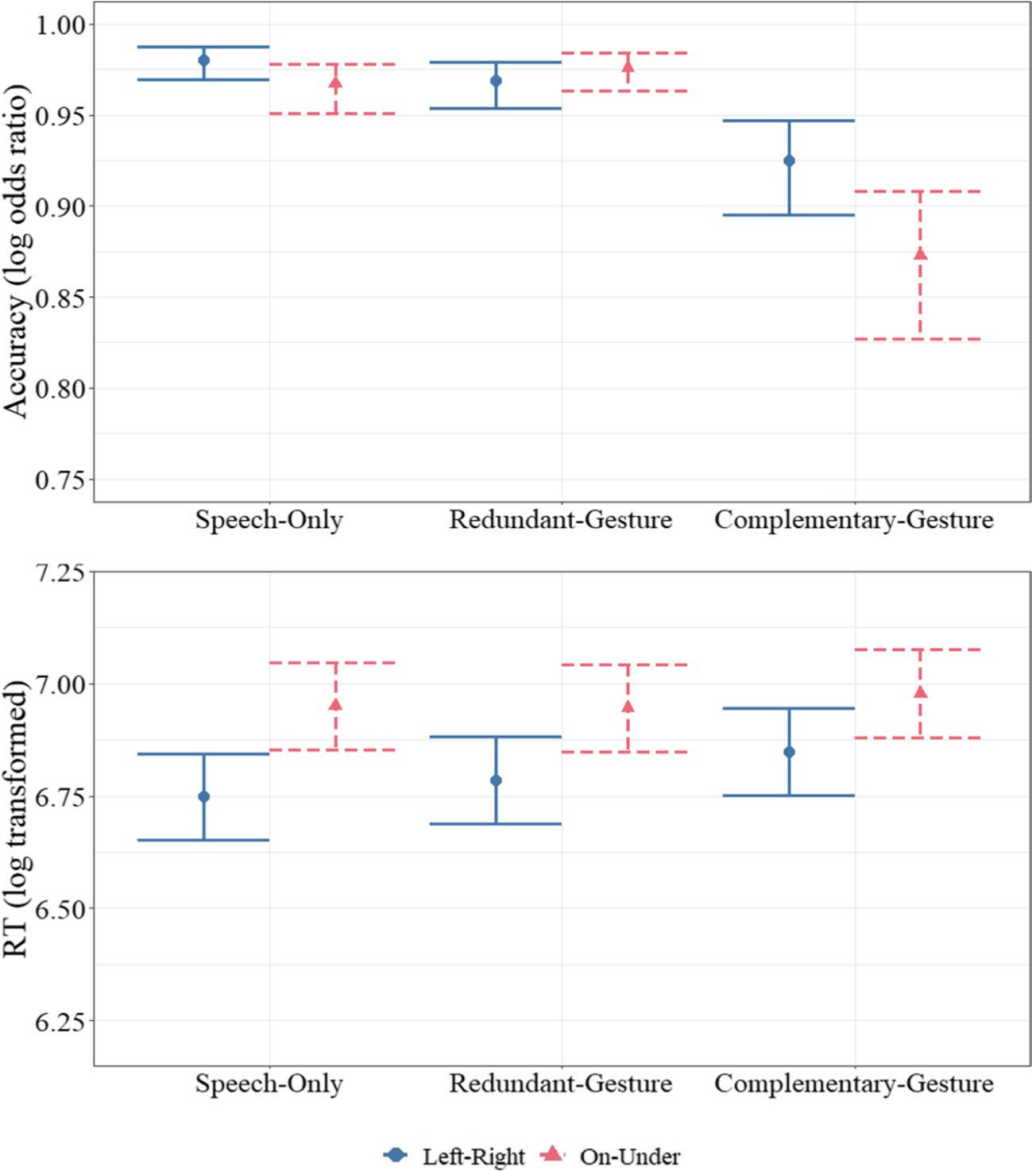
Model estimations for accuracy (top, Model 1a) and reaction times (bottom, Model 1b) across conditions and SR types. The *y*-axes show log odds of accuracy (i.e., correct responding) and log transformed values of RT. The brackets represent 95% confidence intervals

#### Reaction times (Model 1b, Fig. 4)

There was a main effect of the SR type, ${\chi }^{2}$(1) = 41.91, *p* < .001. Across all conditions, participants had longer RTs for on-under trials compared with left-right trials (*β* = 0.16, *SE* = 0.013, *z* = 6.47, *p* < .001).

### Set 2. Corsi span and task performance across conditions and SR types

#### Accuracy (Model 2a, Fig. 5.2a)

There was a three-way interaction among Corsi span, the condition, and the SR type, ${\chi }^{2}$(2) = 6.19, *p* = .04. Higher Corsi spans were associated with higher accuracies in the RG (*β* = 0.61, *SE* = 0.16, *z* = 3.74, *p* < .001) and the CG conditions (*β* = 0.23, *SE* = 0.12, *z* = 1.88, *p* = .05) for left-right trials, and in the CG condition for on-under trials (*β* = 0.28, *SE* = 0.11, *z* = 2.56, *p* = .01).

**Fig. 5:**
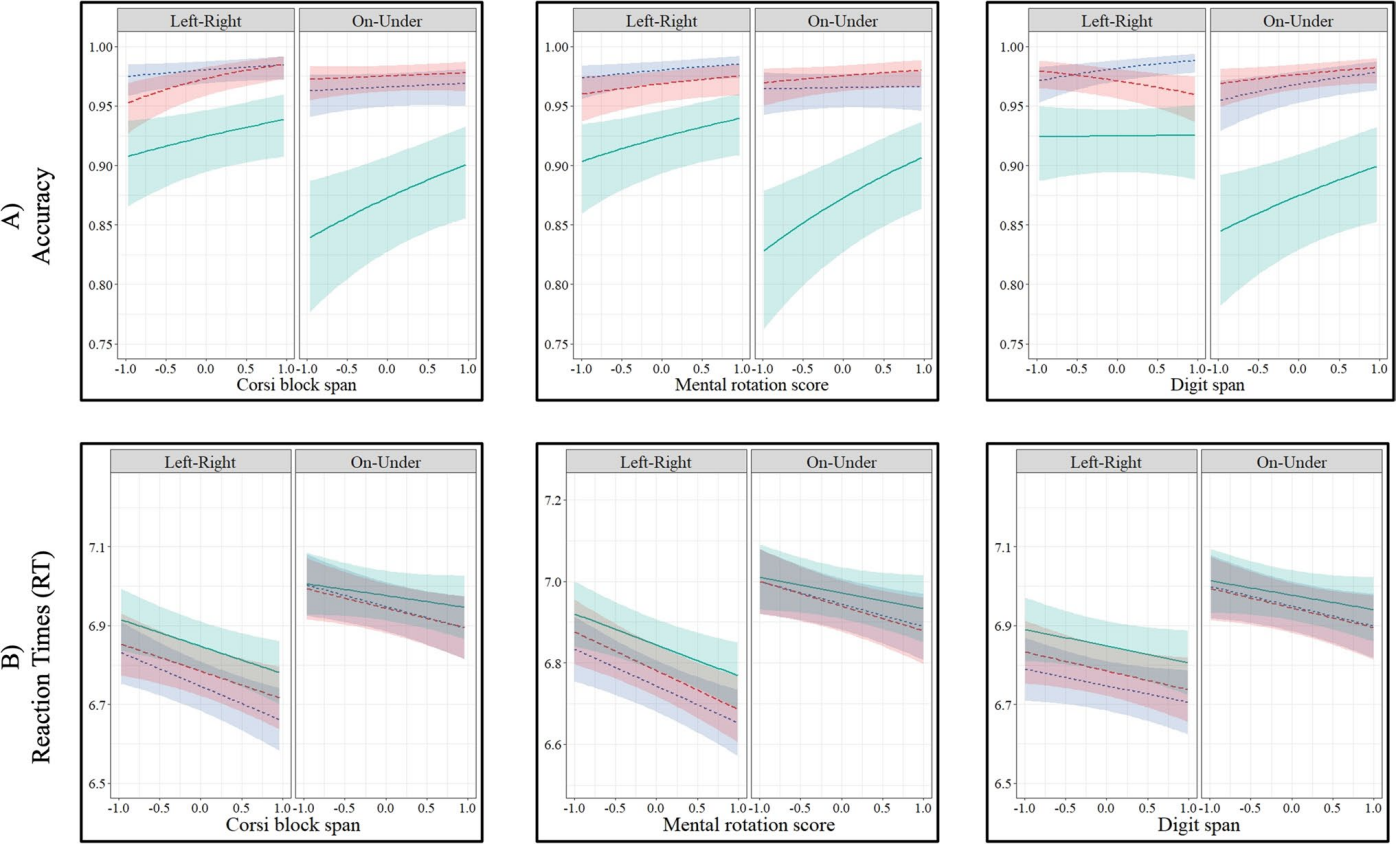
Accuracy (**a**) and reaction times (**b**) as a function of Corsi span (Models 2a and 2b), mental rotation scores (Models 3a and 3b), and digit span (Models 4a and 4b) across conditions and SR types. The *x-*axes show the scaled scores. The* y*-axes show log odds ratio of accuracy and log transformation of RT. The hues around lines represent 95% confidence intervals. (Color figure online)

#### Reaction times (Model 2b, Fig. 5.2b)

There was a significant interaction between Corsi span and the SR type on RT, ${\chi }^{2}$(1) = 9.82, *p* < .01. Across all conditions, higher Corsi spans were associated with shorter RTs only for left-right trials (*β* = −0.08, *SE* = 0.04, *t* = −2.00, *p* = .05), but not for on-under trials (*p* = .23).

### Set 3. Mental rotation and task performance across conditions and SR types

#### Accuracy (Model 3a, Fig. 5.3a)

Mental rotation scores had a significant main effect on the accuracy, ${\chi }^{2}$(1) = 6.17, *p* = .01. Across all conditions and SR types, higher mental rotation scores were associated with higher accuracies (*β* = 0.24, *SE* = 0.10, *z* = 2.48, *p* = .01). The inclusion of the two- and three-way interaction terms did not improve the model (all *p* values > .05).

#### Reaction times (Model 3b, Fig. 5.3b)

There was a significant interaction between mental rotation scores and the SR type, ${\chi }^{2}$(1) = 13.98, *p* < .001. Higher mental rotation scores were associated with shorter RTs only for left-right trials (*β* = −0.09, *SE* = 0.04, *t* = −2.30, *p* = .02), but not for on-under trials (*p* = .18).

### Set 4. Digit span and task performance across conditions and SR types

#### Accuracy (Model 4a, Fig. 5.4a)

There was a significant three-way interaction among digit span, the condition, and the SR type, ${\chi }^{2}$(2) = 7.71, *p* = .02. For on-under trials, higher digit span scores were associated with higher accuracies in all conditions (SO: *β* = 0.39, *SE* = 0.15, *z* = 2.55, *p* = .01; RG: *β* = 0.29, *SE* = 0.16, *z* = 1.81, *p* = .05; CG: *β* = 0.25, *SE* = 0.12, *z* = 2.19, *p* = .03). For left-right trials, higher digit spans were associated with higher accuracies in the SO (*β* = 0.47, *SE* = 0.18, *z* = 2.60, *p* = .01) and lower accuracies in the RG (*β* = −0.36, *SE* = 0.15, *z* = −2.39, *p* = .02).

#### Reaction times (Model 4b, Fig. 5.4b)

None of the terms had a significant effect.

## Discussion

This study examined the role of *spatial* (spatial WM capacity and overall spatial ability) *and verbal skills* (verbal WM capacity) in observing co-speech gestures conveying *redundant versus complementary information* to speech during the comprehension of *left-right versus on-under spatial relations* between objects. Broadly, our results showed that both spatial and verbal skills were related with improved spatial language comprehension as it features spatial information through language medium (both speech and gesture). However, our results also revealed nuanced interactions between these skills and spatial language comprehension, depending on the modality in which the spatial information was expressed. One clear theoretical insight emerged prominently. That is, spatial working memory capacity (measured by Corsi block span task) was associated with accurate comprehension of gestures, particularly when gestures were the sole source of spatial information (i.e., in the CG). This finding highlights the important role of spatial WM in processing visual-spatial information conveyed through gestures, particularly in contexts where gestures provide unique semantic content absent in speech.

Beyond this, our findings showed nuanced interactions among different factors. First, performance was worse when the spatial information was conveyed solely through gesture (CG) compared with when it was available through speech alone (SO) or through both speech and gesture in a bimodal manner (RG), suggesting that visual modality alone might be harder to extract spatial information relative to instances whereby verbal information is available, either alone or alongside gestures. Second, mental rotation ability was related with higher task performance overall, indicating the critical role of object-related dynamic spatial skills for the comprehension of spatial language, particularly featuring relative spatial location of objects. Third, verbal WM capacity was associated with overall task performance, except for a distinct pattern across speech vs. gesture for left-right trials that required viewpoint alignment.

### Task performance

For task performance across conditions, contrary to our predictions, observing redundant gestures along with speech (RG) compared with speech-only (SO) did not enhance comprehension. However, in this context, the accuracy rates were already at the ceiling level in our paradigm, averaging around 96% for the speech-only condition. Hence, there may not be much room for gestural enhancement in the RG condition. Also, in line with our predictions, participants exhibited lower accuracy rates for the CG than others. That is, accuracy was lower when gestures were the sole source for spatial information compared with when the spatial information was given solely in speech or bimodally in both speech and gesture, indicating that when critical spatial information was conveyed unimodally through speech or gestures, listeners tended to rely more on the spoken channel, consistent with the “verbal bias” (Arslan et al., 2024). Our study diverged from some prior research, which suggested that complementary gestures facilitate comprehension more effectively when compared with redundant ones (e.g., Dargue et al., 2021; Yeo et al., 2017). However, we need to consider the specific nature of the gestures used in our study. In our paradigm, complementary gestures primarily served as the sole source of information for task performance, detecting the right spatial relations. They were spatial indicators, providing an indexical reference to a location in space with one hand without conveying fine-tuned detailed information about spatial arrangements between the two objects. This is unlike two-handed placement gestures, where the hands represent objects and their relative spatial relationships, which could be more informative (Karadöller et al., 2021). Overall, listeners might more readily discern the categorical spatial relation (left-right and on-under) from speech compared with gestures that refer to a location indexically (Kranjec et al., 2014).

For task performance across different spatial relation types, contrary to our predictions, participants exhibited lower accuracy rates and slower response times for “on-under” trials compared with “left-right” trials. This finding may be attributed to the kinematic characteristics of the gestures employed in our stimuli. Listeners might have encountered greater difficulty in differentiating “on-under” gestures, which were executed within a smaller gestural space (on the torso) and possessed nuanced kinematic distinctions. These gestures are naturally constrained by the inherent properties of the spatial relations they aim to depict. Spatial distinctions in the “left-right” gestures, on the other hand, were more prominent as they simply indicated locations in the peripheral gestural space These differences, therefore, stem from the natural constraints of the gestures used to represent these spatial relations (see Özer et al., 2023, for a detailed discussion).

### Spatial tasks and gesture processing

In the present study, we have employed two different spatial tasks: the Corsi block span task, which measures visual-spatial WM capacity, and the mental rotation task, which measures overall object-related dynamic spatial skills (Newcombe & Shipley, 2015). Spatial WM capacity was evidenced to play a prominent role in gesture processing (Aldugom et al., 2020; Özer & Göksun, 2020b; Wu & Coulson, 2014) whereas the mental rotation task as a measure of object-related dynamic spatial skills might be important for the overall comprehension of spatial language. Our results indicated a positive relation between mental rotation ability and accuracies in the spatial language comprehension task across all conditions. Evidence suggests a close relationship between spatial transformation abilities and spatial language production (e.g., Balcomb et al., 2011; Pruden et al., 2011). For example, spatial transformation abilities measured through mental transformation task were positively related to children’s preposition (e.g., *behind, under*) production and comprehension (Turan et al., 2021). In line with these, our findings indicate that mental rotation ability, which pertains to dynamic object-related spatial skills, is critical for the overall comprehension of spatial language, particularly when it features relative spatial relations among objects.

Our findings regarding the spatial WM capacity offered a theoretically prominent insight. In line with our prediction, spatial WM capacity was associated with increased accuracy only in cases where spatial descriptions were accompanied by gestures (RG and CG), implying that individuals with higher spatial WM capacities benefited more from observing gestures during comprehension (Aldugom et al., 2020; Özer & Göksun, 2020a, b; Wu & Coulson, 2014). Moreover, spatial WM capacity was particularly important for processing gestures that conveyed essential semantic information not expressed in the accompanying speech. Notably, although the inclusion of the interaction term between mental rotation scores and the modality did not result in a significant improvement, and these results should be interpreted with caution, further slope analyses revealed that mental rotation ability was associated with enhanced comprehension only for the CG condition, with no such relationship observed in the RG condition.^3^ These suggest that spatial skills, particularly spatial WM capacity, play a crucial role for processing gestures that provide unique information that is not readily discernible in speech.

Previous research has shown that spatial-dominant individuals (characterized by higher spatial skills and comparatively lower verbal skills) tend to employ gestures that complement speech with nonredundant information (Abramov et al., 2021; Hostetter & Alibali, 2011). Using gestures can be particularly advantageous for speakers who possess spatial mental representations but may face challenges in effectively conveying those representations through speech alone. Our study builds upon and extends this earlier evidence to the realm of comprehension, revealing that individuals with higher spatial skills may benefit more from observing gestures during comprehension, especially when those gestures convey nonredundant information.

Our results on the relation between spatial tasks and performance across different spatial relation types showed that both the Corsi block span task and the mental rotation task were related to faster responses only for left-right relations, with no such relationship observed for on-under trials. “Left-right” spatial relations typically require viewpoint alignment between conversational partners, thereby imposing more significant processing demands on their spatial cognitive capabilities (Galati & Avraamides, 2013; Galati et al., 2013). This reliance on perspective-taking processes might explain the observed relationship between spatial skills and reaction times for left-right trials. In contrast, we did not find any relationship between spatial skills and the comprehension of on-under trials. These results might reflect a key difference in the cognitive demands associated with the two spatial relations. Although on-under gestures were harder compared with left-right gestures, which was evident in longer RTs and lower accuracies for on-under trials overall, this might be attributed to the inherent perceptual ambiguity of these gestures rather than a greater cognitive difficulty. On-under gestures, executed within a smaller gestural space with nuanced kinematic distinctions, likely introduce perceptual challenges. Left-right gestures, on the other hand, might be more cognitively demanding due to the involvement of perspective-taking processes, which could explain why the comprehension left-right trials present a relationship with Corsi scores. Spatial skills, which encompass a range of skills related to processing spatial information, play a pivotal role in comprehending spatial language, particularly for spatial terms that require visual-spatial perspective-taking. Current findings highlight the relation between spatial skills and the comprehension of spatial language across different spatial relation categories associated with varying cognitive demands.

### Verbal WM task and gesture comprehension

Our results revealed an interesting link between verbal WM capacity and processing gestures. Previous studies suggested a role for verbal skills in gesture processing particularly when verbal resources are required to interpret the referent of gestures (e.g., Momsen et al., 2020; Schubotz et al., 2021). As gestures in the current study do not necessarily require the accompanying speech for their interpretation as they indexically show a particular location in speech, we expected to observe an effect of verbal WM capacity on comprehension for instances in which critical spatial relation has been expressed through speech (SO and RG). Contrary to our expectations, verbal WM was associated with enhanced comprehension across all conditions for on-under trials. For left-right trials, on the other hand, verbal WM was related to enhanced comprehension for the SO condition and impaired comprehension for the RG condition. This implies that individuals with higher verbal skills might tend to rely heavily on the spoken channel, and processing additional visual information, particularly ones that require visual perspective-taking (i.e., left-right), might impede their comprehension (Hostetter et al., 2018).

## Limitations and future research

The current study has some limitations which would open avenues for future research. First, the ceiling effect observed in the task (particularly in the SO and the RG conditions) may limit the generalizability of the findings by potentially obscuring the relations among spatial and verbal skills and gesture processing. However, despite this limitation, we still found significant effects of spatial and verbal skills on task performance, highlighting the robustness of these relationships. Future studies could benefit from using language comprehension tasks with a greater difficulty to further explore this relationship. Second, while the present study employed spatial and verbal tasks that are closely tied to gesture processing in prior research (Momsen et al., 2021; Özer & Göksun, 2020b; Wu & Coulson, 2014), future research could employ different tasks, such as the operation span task or the reading span task that are well-established measures of language comprehension and assess complex working memory incorporating the maintenance and manipulation of information, to provide additional insights for the interplay between spatial and verbal skills and gesture processing. Third, future research could extend the current examination to other languages and cultures (Azar et al., 2020; Kita, 2009), different spatial relations such as ones that feature containment and support (Landau et al., 2017), contexts where gestures are used with nonspatial abstract sentences (Nagels et al., 2013; Steines et al., 2021), and a broader range of different cognitive correlates of gesture processing (Nagels et al., 2015).

## Conclusion

In conclusion, our study suggests that the effects of gestures on comprehension can be influenced by various cognitive and contextual factors, including listener’s cognitive skills, the semantic relation between gesture and speech, and the type and the complexity of the spatial relation. The findings notably emphasized the critical role of spatial working memory in processing gestures, particularly when these gestures convey essential semantic information that could not be extracted from the accompanying speech. This study provides valuable insights into the intricate interplay between cognitive skills and semantic properties of gestures in determining the role of gestures in language comprehension, ultimately shedding light on the complex dynamics of multimodal language and cognition.

## Supplementary information

Below is the link to the electronic supplementary material.

Supplementary file1 (DOCX 168 KB)

## Data Availability

The data presented in the current manuscript and a sample of video stimuli can be found online in the Open Science Framework repository (view-only link): https://osf.io/d6xzw/?view_only=200bb98a9437414cbd6dda73967a9e6b

## Footnotes

1 There are several other types of gestures. *Deictic gestures* refer to entities in the immediate environment by pointing, *metaphoric gestures* represent abstract concepts, and *beat gestures* are rhythmical hand movements that are time-locked to the prosody of speech without carrying a propositional content (McNeill, 1992). However, as stated, the current study particularly focuses on *iconic gestures* that imagistically represent concrete entities, such as actions, events, and objects.

2 It is important to note that the definition of mismatching gestures in this study was different from other studies that conceptualized mismatching gestures as those that express complementary information to the speech (e.g., a child saying “the dish is lower than the glass” and at the same time producing a wider C hand near the dish and a narrower C hand near the glass to express varying widths of these containers when describing the solutions of liquid conservation; Church & Goldin-Meadow, 1986; Goldin-Meadow et al., 1993). Contrary to this conceptualization, mismatching gestures in Özer and Göksun (2020b) expressed conflicting information to speech (e.g., saying “cut” and gesturing “turn”).

3 The inclusion of the two- and three-way interaction terms among the mental rotation scores, the condition, and the spatial relation type did not improve the model, *p* values > .05. However, simple slope analyses for each level of the condition and the SR type suggested that mental rotation scores were associated with increased accuracies only for the CG (left-right: *β* = 0.26, *SE* = 0.12, *z* = 2.17, *p* = .03; on-under: *β* = 0.36, *SE* = 0.11, *z* = 3.24, *p* < .01).

## Code availability

The R program script to perform the analyses in the current manuscript can be found online in the Open Science Framework repository (view-only link):

https://osf.io/d6xzw/?view_only=200bb98a9437414cbd6dda73967a9e6b
