# Supplementary material for: Spatial working memory is critical for gesture processing: Evidence from gestures with varying semantic links to speech
Source: Psychon Bull Rev. 2025 Feb 10;32(4):1639–53. doi: 10.3758/s13423-025-02642-4 (PMC12325567; doi:10.3758/s13423-025-02642-4)
Supplement: Supplementary file 1 — Supplementary file1 (DOCX 168 KB) [file 13423_2025_2642_MOESM1_ESM.docx]

**Supplementary Materials**

***Individual Differences Measures***

Table 1 presents descriptive statistics for raw scores of Corsi span, digit span, and mental rotation scores (i.e., average percentage of accuracy across all trials). Figure 1 shows the distribution of raw scores across individual differences measures. The bivariate correlations among individual differences measures were as follows: Corsi span and mental rotation score (*r* = .33, *p* < .01), Corsi span and digit span (*r* = .28, *p* < .05), mental rotation score and digit span (*r* = .23, *p* = .06).

**Table1.**

Descriptive statistics for raw scores in individual differences tasks

|  | ***Corsi span*** | | | ***Mental rotation*** | | | | ***Digit span*** |  |  |
| --- | --- | --- | --- | --- | --- | --- | --- | --- | --- | --- |
| *Mean* |  | 6.47 |  | | 0.80 |  | 7.07 | | |  |
| *SE* |  | 0.15 |  | | 0.01 |  | 0.16 | | |  |
| *Median* |  | 7.00 |  | | 0.83 |  | 7.00 | | |  |
| *SD* |  | 1.26 |  | | 0.12 |  | 1.37 | | |  |
| *Variance* |  | 1.59 |  | | 0.01 |  | 1.86 | | |  |
| *Min. – Max.* |  | 4-9 |  | | .51-.98 |  | 5-10 | | |  |
| *Skewness* |  | 0.11 |  | | -0.67 |  | 0.36 | | |  |
| *SD Skewness* |  | 0.29 |  | | 0.29 |  | 0.29 | | |  |
| *Kurtosis* |  | -0.75 |  | | -0.40 |  | -0.46 | | |  |
| *SD Kurtosis* |  | 0.57 |  | | 0.57 |  | 0.57 | | |  |

Notes: *SE*= Standard error of the mean, *SD*= standard deviation

**Figure 1.**


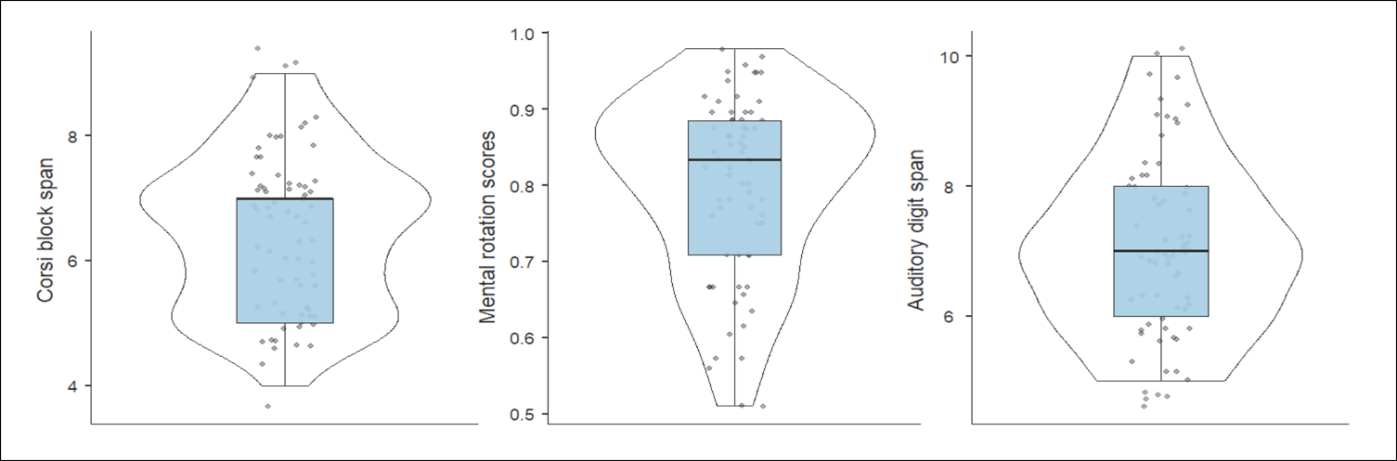
The distribution of raw scores across individual differences measures

***Main Analyses - Mixed Effects Models***

Tables 2 to 5 display parameter estimates and Type-III Wald Chi-Square test results across two outcome variables (accuracy and RT) separately for each model. We used sum-to-zero contrast coding. In parameter estimates tables, intercepts refer to the grand mean and each contrast encoded the deviation from the intercept for a given factor. The parameter estimates denote log odds ratio for the accuracy and the log transformed RTs for RT models.

**Table 2.**

Parameter estimates and Type-III Wald Chi-Square test results for Models 1a and 1b. The models in this table test how accuracy (Model 1a) and RT (Model 1b) changed across conditions and SR types.

| **MODEL PARAMETER ESTIMATES** | | | | | | | | | |
| --- | --- | --- | --- | --- | --- | --- | --- | --- | --- |
|  | **Accuracy** | | | |  | **Reaction Times** | | | |
|  | *β* | *SE* | *z* | *p* |  | *β* | *SE* | *t* | *p* |
| FIXED EFFECTS |  |  |  |  |  |  |  |  |  |
| Intercept | 3.15 | 0.12 | 25.69 | < .001 |  | 6.88 | 0.04 | 169.34 | <.001 |
| Condition (= SO) | 0.50 | 0.11 | 4.45 | <.001 |  | -0.03 | 0.02 | -1.52 | .13 |
| Condition (= RG) | 0.43 | 0.11 | 3.86 | <.001 |  | -0.01 | 0.02 | -0.58 | .56 |
| SR Type (= L-R) | 0.14 | 0.08 | 1.83 | .07 |  | -0.08 | 0.01 | -6.47 | <.001 |
| SO * L-R | 0.12 | 0.11 | 1.09 | .27 |  | -0.02 | 0.02 | -1.07 | .28 |
| RG * L-R | -0.28 | 0.11 | -2.49 | .01 |  | 0.00 | 0.02 | 0.11 | .91 |
|  | *Var* | *SD* |  |  |  | *Var* | *SD* |  |  |
| RANDOM EFFECTS |  |  |  |  |  |  |  |  |  |
| Subject-Intercept | 0.55 | 0.74 |  |  |  | 0.10 | 0.32 |  |  |
| Item-Intercept | 0.38 | 0.61 |  |  |  | 0.02 | 0.13 |  |  |
|  |  |  |  |  |  |  |  |  |  |
| **ANALYSIS OF DEVIANCE TABLE (TYPE-III WALD CHISQUARE TESTS)** | | | | | | | | | |
|  | *ꭓ^2^* | *df* | *p* |  |  | *ꭓ^2^* | *df* | *p* |  |
| Intercept | 659.77 | 1 | <.001 |  |  | 28,676 | 1 | <.001 |  |
| Condition | 82.87 | 2 | <.001 |  |  | 4.67 | 2 | .09 |  |
| SR Type | 3.37 | 1 | .06 |  |  | 41.91 | 1 | <.001 |  |
| Condition * SR Type | 6.43 | 2 | .04 |  |  | 1.38 | 2 | .50 |  |

**Note:** SO = Speech-Only, RG = Redundant-Gesture, SR Type = spatial relation type, L-R= Left-Right, Var = variance, SE= standard error, SD= standard deviation

**Table 3.**

Parameter estimates and Type-III Wald Chi-Square test results for Models 2a and 2b. The models in this table test how accuracy (Model 2a) and RT (Model 2b) changed as a function of Corsi block scores across conditions and SR types.

| **MODEL PARAMETER ESTIMATES** | | | | | | | | | |
| --- | --- | --- | --- | --- | --- | --- | --- | --- | --- |
|  | **Accuracy** | | | |  | **Reaction Times** | | | |
|  | *β* | *SE* | *z* | *p* |  | *β* | *SE* | *t* | *p* |
| FIXED EFFECTS |  |  |  |  |  |  |  |  |  |
| Intercept | 3.16 | 0.12 | 26.73 | < .001 |  | 6.87 | 3.99 | 172.16 | <.001 |
| Condition (= SO) | 0.47 | 0.11 | 4.10 | <.001 |  | -2.72 | 1.78 | -1.53 | .13 |
| Condition (= RG) | 0.47 | 0.11 | 4.14 | <.001 |  | -1.02 | 1.78 | -0.57 | .57 |
| SR Type (= L-R) | 0.17 | 0.08 | 2.23 | .03 |  | -8.17 | 1.26 | -6.47 | <.001 |
| Corsi span | 0.26 | 0.10 | 2.72 | .01 |  | -6.11 | 3.76 | -1.62 | .11 |
| SO * L-R | 0.10 | 0.11 | 0.91 | .37 |  | -1.93 | 1.78 | -1.08 | .28 |
| RG * L-R | -0.22 | 0.11 | -1.90 | .06 |  | 1.93 | 1.78 | 0.11 | .91 |
| SO * Corsi | -0.09 | 0.07 | -1.22 | .22 |  | -1.11 | 6.58 | -1.69 | .09 |
| RG * Corsi | 0.10 | 0.08 | 1.32 | .19 |  | 1.85 | 6.59 | 0.03 | .98 |
| L-R * Corsi | 0.10 | 0.05 | 2.02 | .04 |  | -1.49 | 4.74 | -3.13 | <.01 |
| SO * L-R * Corsi | -0.02 | 0.07 | -0.30 | .76 |  | -9.10 | 6.57 | -0.14 | .89 |
| RG * L-R * Corsi | 0.15 | 0.07 | 2.03 | .04 |  | 5.26 | 6.60 | 0.80 | .42 |
|  | *Var* | *SD* |  |  |  | *Var* | *SD* |  |  |
| RANDOM EFFECTS |  |  |  |  |  |  |  |  |  |
| Subject-Intercept | 0.47 | 0.69 |  |  |  | 0.10 | 0.32 |  |  |
| Item-Intercept | 0.38 | 0.61 |  |  |  | 0.02 | 0.13 |  |  |
|  |  |  |  |  |  |  |  |  |  |
| **ANALYSIS OF DEVIANCE TABLE (TYPE-III WALD CHISQUARE TESTS)** | | | | | | | | | |
|  | *ꭓ^2^* | *df* | *p* |  |  | *ꭓ^2^* | *df* | *p* |  |
| Intercept | 714.6 | 1 | <.001 |  |  | 29,640 | 1 | <.001 |  |
| Condition | 83.28 | 2 | <.001 |  |  | 4.67 | 2 | .10 |  |
| SR Type | 4.99 | 1 | .02 |  |  | 41.93 | 1 | <.001 |  |
| Corsi span | 7.38 | 1 | <.01 |  |  | 2.63 | 1 | .10 |  |
| Condition*SR Type | 3.68 | 2 | .16 |  |  | 1.42 | 2 | .49 |  |
| Condition*Corsi | 1.95 | 2 | .38 |  |  | 3.52 | 2 | .17 |  |
| SR Type*Corsi | 4.08 | 1 | .04 |  |  | 9.82 | 1 | <.01 |  |
| Condition*SR Type*Corsi | 6.19 | 2 | .04 |  |  | 0.70 | 22 | .71 |  |

**Note:** SO = Speech-Only, RG = Redundant-Gesture, SR Type = spatial relation type, L-R= Left-Right, Corsi = Corsi span scores, Var = variance, SE= standard error, SD= standard deviation

**Table 4.**

Parameter estimates and Type-III Wald Chi-Square test results for Models 3a and 3b. The models in this table test how accuracy (Model 3a) and RT (Model 3b) changed as a function of mental rotation scores across conditions and SR types.

| **MODEL PARAMETER ESTIMATES** | | | | | | | | | |
| --- | --- | --- | --- | --- | --- | --- | --- | --- | --- |
|  | **Accuracy** | | | |  | **Reaction Times** | | | |
|  | *β* | *SE* | *z* | *p* |  | *β* | *SE* | *t* | *p* |
| FIXED EFFECTS |  |  |  |  |  |  |  |  |  |
| Intercept | 3.13 | 0.12 | 26.52 | < .001 |  | 6.87 | 4.02 | 171.03 | <.001 |
| Condition (= SO) | 0.49 | 0.11 | 4.30 | <.001 |  | -2.69 | 1.79 | -1.51 | .13 |
| Condition (= RG) | 0.43 | 0.11 | 3.83 | <.001 |  | -1.04 | 1.79 | -0.58 | .56 |
| SR Type (= L-R) | 0.15 | 0.08 | 1.92 | .05 |  | -8.12 | 1.27 | -6.41 | <.001 |
| Mental Rotation (MR) | 0.24 | 0.10 | 2.48 | .01 |  | -6.91 | 3.78 | -1.84 | .07 |
| SO * L-R | 0.14 | 0.11 | 1.21 | .22 |  | -1.93 | 1.79 | -1.08 | .28 |
| RG * L-R | -0.27 | 0.11 | -2.43 | .01 |  | 1.95 | 1.79 | 0.11 | .91 |
| SO * MR | -0.07 | 0.07 | -0.98 | .32 |  | -3.71 | 6.65 | -0.56 | .58 |
| RG * MR | 0.00 | 0.07 | 0.02 | .98 |  | -8.45 | 6.66 | -1.27 | .20 |
| L-R * MR | 0.03 | 0.05 | 0.73 | .46 |  | -1.79 | 4.80 | -3.74 | <.001 |
| SO * L-R * MR | 0.10 | 0.07 | 1.42 | .15 |  | 1.88 | 6.64 | 0.03 | .98 |
| RG * L-R * MR | -0.02 | 0.07 | -0.27 | .79 |  | 5.90 | 6.66 | 0.09 | .93 |
|  | *Var* | *SD* |  |  |  | *Var* | *SD* |  |  |
| RANDOM EFFECTS |  |  |  |  |  |  |  |  |  |
| Subject-Intercept | 0.47 | 0.69 |  |  |  | 0.10 | 0.32 |  |  |
| Item-Intercept | 0.38 | 0.61 |  |  |  | 0.02 | 0.13 |  |  |
|  |  |  |  |  |  |  |  |  |  |
| **ANALYSIS OF DEVIANCE TABLE (TYPE-III WALD CHISQUARE TESTS)** | | | | | | | | | |
|  | *ꭓ^2^* | *df* | *p* |  |  | *ꭓ^2^* | *df* | *p* |  |
| Intercept | 703.2 | 1 | <.001 |  |  | 29,252 | 1 | <.001 |  |
| Condition | 79.88 | 2 | <.001 |  |  | 4.60 | 2 | .10 |  |
| SR Type | 3.68 | 1 | .05 |  |  | 41.16 | 1 | <.001 |  |
| Mental Rotation (MR) | 6.17 | 1 | .01 |  |  | 3.39 | 1 | .07 |  |
| Condition*SR Type | 5.99 | 2 | .05 |  |  | 1.41 | 2 | .49 |  |
| Condition*MR | 1.72 | 2 | .42 |  |  | 3.15 | 2 | .21 |  |
| SR Type*MR | 0.54 | 1 | .46 |  |  | 13.98 | 1 | <.001 |  |
| Condition*SR Type*MR | 2.91 | 2 | .23 |  |  | 0.01 | 22 | .99 |  |

**Note:** SO = Speech-Only, RG = Redundant-Gesture, SR Type = spatial relation type, L-R= Left-Right, MR = mental rotation scores, Var = variance, SE= standard error, SD= standard deviation

**Table 5.**

Parameter estimates and Type-III Wald Chi-Square test results for Models 4a and 4b. The models in this table test how accuracy (Model 4a) and RT (Model 4b) changed as a function of digit span scores across conditions and SR types.

| **MODEL PARAMETER ESTIMATES** | | | | | | | | | |
| --- | --- | --- | --- | --- | --- | --- | --- | --- | --- |
|  | **Accuracy** | | | |  | **Reaction Times** | | | |
|  | *β* | *SE* | *z* | *p* |  | *β* | *SE* | *t* | *p* |
| FIXED EFFECTS |  |  |  |  |  |  |  |  |  |
| Intercept | 3.18 | 0.12 | 26.05 | < .001 |  | 6.87 | 4.02 | 170.85 | <.001 |
| Condition (= SO) | 0.52 | 0.11 | 4.50 | <.001 |  | -2.70 | 1.78 | -1.52 | .13 |
| Condition (= RG) | 0.44 | 0.11 | 3.90 | <.001 |  | -1.04 | 1.78 | -0.58 | .56 |
| SR Type (= L-R) | 0.15 | 0.08 | 1.93 | .05 |  | -8.18 | 1.26 | -6.47 | <.001 |
| Digit span (Digit) | 0.17 | 0.10 | 1.72 | .09 |  | -4.50 | 3.83 | -1.17 | .24 |
| SO * L-R | 0.12 | 0.11 | 1.08 | .29 |  | -1.92 | 1.78 | -1.07 | .28 |
| RG * L-R | -0.26 | 0.11 | -2.26 | .02 |  | 1.93 | 1.78 | 0.11 | .91 |
| SO * Digit | 0.25 | 0.07 | 3.53 | <.001 |  | -1.25 | 6.58 | -0.19 | .85 |
| RG * Digit | -0.21 | 0.07 | -2.91 | <.01 |  | -4.06 | 6.62 | -0.61 | .54 |
| L-R * Digit | -0.14 | 0.05 | -2.82 | <.01 |  | 6.51 | 4.75 | 0.14 | .89 |
| SO * L-R * Digit | 0.18 | 0.07 | 2.36 | .02 |  | 3.44 | 6.57 | 0.52 | .60 |
| RG * L-R * Digit | -0.19 | 0.07 | -2.67 | <.01 |  | 1.40 | 6.62 | 0.02 | .98 |
|  | *Var* | *SD* |  |  |  | *Var* | *SD* |  |  |
| RANDOM EFFECTS |  |  |  |  |  |  |  |  |  |
| Subject-Intercept | 0.53 | 0.73 |  |  |  | 0.10 | 0.32 |  |  |
| Item-Intercept | 0.38 | 0.62 |  |  |  | 0.02 | 0.13 |  |  |
|  |  |  |  |  |  |  |  |  |  |
| **ANALYSIS OF DEVIANCE TABLE (TYPE-III WALD CHISQUARE TESTS)** | | | | | | | | | |
|  | *ꭓ^2^* | *df* | *p* |  |  | *ꭓ^2^* | *df* | *p* |  |
| Intercept | 678.59 | 1 | <.001 |  |  | 29,192 | 1 | <.001 |  |
| Condition | 86.25 | 2 | <.001 |  |  | 4.65 | 2 | .10 |  |
| SR Type | 3.71 | 1 | .05 |  |  | 41.87 | 1 | <.001 |  |
| Digit Span (Digit) | 2.94 | 1 | .09 |  |  | 1.38 | 1 | .24 |  |
| Condition*SR Type | 5.23 | 2 | .07 |  |  | 1.39 | 2 | .50 |  |
| Condition*Digit | 12.03 | 2 | <.01 |  |  | 0.65 | 2 | .72 |  |
| SR Type*Digit | 7.93 | 1 | <.01 |  |  | 0.02 | 1 | .89 |  |
| Condition*SR Type*Digit | 7.71 | 2 | .02 |  |  | 0.36 | 2 | .83 |  |

**Note:** SO = Speech-Only, RG = Redundant-Gesture, SR Type = spatial relation type, L-R= Left-Right, Digit = digit span scores, Var = variance, SE= standard error, SD= standard deviation
